# Adaptive Belief Rule Base Modeling of Complex Industrial Systems Based on Sigmoid Functions

**DOI:** 10.3390/e27111157

**Published:** 2025-11-14

**Authors:** Haolan Huang, Shucheng Feng, Jingying Li, Tianshu Guan, Hailong Zhu

**Affiliations:** 1 The School of Computer Science and Information Engineering, Harbin Normal University, Harbin 150025, China; 2 The School of Software, Dalian University of Foreign Languages, Dalian 116044, China

**Keywords:** complex industrial systems, nonlinear belief rule base, evidential reasoning

## Abstract

In response to the challenges posed by multifactorial nonlinear relationships and uncertainties, and to address the limitations of the existing Belief Rule Base (BRB) in nonlinear fitting, uncertainty representation, and parameter optimization, this paper presents an improved reliable modeling method using a nonlinear belief rule base (R-NBRB). First, the linear inference mechanism is replaced by a smooth nonlinear S-function. This replacement better adapts to nonlinear dynamics in complex industrial systems. Second, attribute reliability is quantified through a reliability assessment method. Data, reliability, and expert knowledge are integrated using the Evidential Reasoning (ER) algorithm. Uncertainty is expressed in the form of belief degrees. Finally, the Covariance Matrix Adaptation Evolution Strategy (CMA-ES) algorithm is applied to optimize the inference parameters. Decision bias caused by insufficient expert knowledge is thereby reduced. Experiments were conducted on a task involving the detection of a petroleum pipeline leak. The mean squared error (MSE) of the R-NBRB model is only 0.2569. This represents a 28.24% reduction compared with the BRB model. The proposed method’s effectiveness and adaptability in complex industrial situations are confirmed.

## 1. Introduction

Complex industrial systems often show nonlinear dynamics and operate in uncertain environments. They also face practical challenges like sensor noise, equipment wear, and external disturbances [1]. As modern industrial systems continue to grow in scale and structural complexity, traditional modeling methods face significant limitations in addressing nonlinear dynamic responses and uncertain factors. Therefore, in critical fields such as industrial automation, smart manufacturing, and energy transportation, there is an urgent need to develop new models that integrate reliability and generalizability to accurately describe their dynamic characteristics. This has become a crucial requirement and a fundamental basis for enhancing operational safety and achieving precise control and intelligent decision-making [2].

The modeling process for complex industrial systems focuses on reliably assessing system states. Its outputs help with intelligent judgement and decision support in operational environments. In the context of complex systems, the term “decision-making” specifically refers to the model’s ability to discriminate and reason about system operational states under uncertain conditions. This capability gives a solid basis for tasks like risk warnings and maintenance strategies. It acts as a key link between system monitoring and operational actions. Thus, the decision-making discussed in this study emphasizes intelligent inference on the basis of state assessment of complex industrial systems, reflecting a complete technical chain from data perception to operational decision-making [3]. This theory matches the needs of state cognition in complex systems. It also shows the practical benefits of intelligent modeling methods in industrial operations and maintenance.

Currently, the research methods in this field can be divided into three main categories: physics model-driven, data-driven, and hybrid-driven models.

(1) Physics model-driven model

The physics model-driven model is a methodology. It builds models using physical laws and mechanisms. Then, it analyses the system and makes predictions. Its core does not rely on data analysis. It describes the internal operation mechanism of the system through mathematical equations. A fuzzy model-based observer was proposed by Baigzadehnoe et al. [4] to handle the fault detection problem of nonlinear networked control systems. A physics-of-failure model including side reactions was established by Li et al. [5]. The parameter evolution of the model is analyzed to construct fault boundaries. The effective diagnosis of multiple internal aging faults in lithium-ion batteries is realized in this way. Choudhury et al. proposed a fault diagnosis approach that integrates the squared envelope spectrum of bearing vibration signals with a pre-trained convolutional neural network [6]. This method allows for the effective extraction of second-order cyclostationary features via 2D image representation, enabling efficient fault diagnosis. However, physics models usually ignore the statistical laws or empirical knowledge hidden in historical data. They rely only on equation derivation.

(2) Data-driven model

Data-driven models can operate without relying heavily on prior mechanisms [7]. They mine the operation laws of complex industrial systems from data by analyzing large amounts of data. Excessive dependence on the internal mechanisms of systems is avoided. For example, a data-driven hybrid power system framework was proposed by Wu et al. [8]. Noise reduction via generative adversarial networks and residual learning technology are applied in this framework. High-precision dynamic modeling of single-cell transcriptome data is realized. The dependence on prior knowledge of traditional mechanism models is significantly reduced. The problem of high data demand caused by excessive parameters of data-driven models was addressed by Chattopadhyay et al. [9]. A stochastic model based on a convolutional variational autoencoder is proposed. This model is superior to traditional deterministic models in short-term prediction ability. Research on machine learning and deep learning algorithms was conducted by Soleimani et al. [10]. A new type of data-driven prediction model has been developed. Modeling of the degradation of complex industrial systems, fault detection, and prediction of remaining useful life are realized via this model. However, the accuracy of this method generally relies on the precision and completeness of the data collected from complex industrial systems. Consequently, these methods often struggle to handle system modeling processes with small-sample datasets in complex environments.

(3) Hybrid-driven models

The hybrid-driven modeling approach integrates physical mechanisms with data algorithms, maintaining model interpretability while significantly enhancing modeling efficiency and prediction accuracy, making it particularly suitable for the simulation and optimization of complex industrial systems. A hybrid knowledge system method was proposed by Yuan et al. [11]. This method constructs and performs reasoning within distinct expert knowledge systems, tailored to the specific categories of accessible information. A hybrid model combining gear dynamics equations and a multi-dimensional BRB was designed by Gao et al. [12]. The rule base is initialized via a data-driven approach in this model. Excellent performance is exhibited by the model. The development of a novel Graph Convolutional Network (GCN)-based fault diagnosis technique was presented by Chen’s research team [13]. This method combines available measurements and prior knowledge.

The Belief Rule Base (BRB) is an uncertain hybrid modeling method that can effectively combine quantitative information and expert knowledge [14]. Uncertainty is described in the form of a belief distribution. It has good adaptability to small-sample data. It reduces the dependence of modeling on large-scale data, giving it wide application value in complex industrial system modeling. Currently, the BRB model has been widely used in the modeling process of complex industrial systems [15]. A model that combines interval addition and interpretability constraints was proposed by He et al. [16]. The rule base parameters are optimized via a data-driven approach in this model. Moreover, the traceability of the physics model is retained. A new model based on BRB was proposed by Lian et al. [17]. This model includes sensor disturbances to improve the accuracy and reliability of performance assessments in complex industrial systems. Zhang et al. introduced a fault diagnosis model based on a Micro-Belief Rule Base (MBRB) for complex electromechanical systems [18]. This model is constructed via an enhanced belief rule architecture.

Despite the significant advantages of the BRB method, some challenges in its practical application still need to be addressed. The BRB method uses linear functions for model reasoning [19]. It cannot effectively handle the complex nonlinear relationships between input features and outputs [20]. Moreover, complex industrial systems are highly susceptible to various disturbances in real-world environments and are characterized by substantial uncertainties [21]. The BRB model has constraints in managing such uncertainties and often struggles to address intricate scenarios.

In response to these challenges, the key innovations of the reliable modeling method using a nonlinear belief rule base (R-NBRB) model introduced in this study are outlined below:

(1) The model’s reasoning mechanism employs a smoothly varying S-function to adaptively calibrate the matching degree. The corresponding matching degrees can also produce relatively significant differences. Thus, the degrees of activation of different rules are distinguished more accurately. An optimization algorithm fine-tunes the expert knowledge in the improved S-function and the parameters created during the model reasoning process. The method provides dual assurance of model interpretability and uncertainty reduction.
(2) By embedding attribute reliability into the modeling framework, the proposed method effectively suppresses uncertainties arising from external complex environments during the inference process.
(3) When applied to the representative complex industrial task of petroleum pipeline leak detection, the proposed model not only significantly improves the accuracy of leak identification and decision-making, but also provides a solid foundation for highly reliable decision support, thereby comprehensively enhancing the safety and trustworthiness of system operations.

The structure of this paper is as follows. In Section 2, the problems existing in the complex industrial system modeling process of the R-NBRB model are identified and solved. In Section 3, the reasoning process of the R-NBRB model is explained in detail. In Section 4, a comprehensive analysis of the R-NBRB model is conducted through the case of oil pipeline leakage. Finally, in Section 5, the research content is summarized.

## 2. Problem Description

Several fundamental challenges emerge when the R-NBRB framework is employed in complex industrial system modeling:

(1) How can complex nonlinear relationships between input features and outputs be handled?

In the BRB model, the linear functions used for reasoning struggle to capture the complex and tightly linked relationships between inputs and outputs [22]. Underfitting, insufficient generalization ability, and low characterization accuracy tend to occur. To address this issue, this paper adjusts the model matching degree calculation process by incorporating S-functions. Input data and expert knowledge are effectively represented. Model decision accuracy is improved. Complex nonlinear relationships between inputs and outputs are handled efficiently. The reasoning process can be expressed by Equation (1).

(1) $${\alpha \text{\_}s}_{i,j}=S(\Omega_{Expert},\Omega_{D})$$

where set $\Omega_{Expert}$, which contains all the expert knowledge used in the BRB model reasoning process, is defined. Set $\Omega_{D}$, which represents all the input data required for the decision-making process of complex industrial systems, is specified. The updated matching degree ${\alpha \text{\_}s}_{i,j}$, which is obtained when the model is constructed via nonlinear functions, is denoted.

(2) How to adapt an S-function to the complex nonlinear relationships between input features and outputs.

The traditional S-function has a fixed mapping function, making it difficult to adapt to complex nonlinear correlations such as multimodality and strong coupling [23]. Its parameters lack adaptability to changes in nonlinear relationships. To tackle these limitations, this paper adds a nonlinear operator. It comes from expert knowledge and is designed for the specific context of the complex industrial system within the S-function framework. The model’s ability lies in accurately characterizing the sophisticated nonlinear input-output relationships inherent to complex industrial system modeling. Moreover, an optimization algorithm is used to adjust the nonlinear operator given by the experts. This operator supports adaptive and dynamic updates during model reasoning, thereby preserving interpretability while concurrently enhancing the precision of the inference process. The adaptive adjustment process of the nonlinear operator within the complex industrial system modeling framework is expressed in Equation (2).

(2) $$a\text{\_}param=C(a_{expert},\Omega )$$

where C(•) denotes the optimization function, $a_{expert}$ represents the nonlinear operator provided by experts based on the initial state of the complex industrial system, Ω specifies the parameter set to be optimized during the model reasoning process, and a_param represents the nonlinear operator adaptively adjusted by the optimization function in the model reasoning process, which is utilized for the adaptive update of the complex nonlinear fitting between input features and outputs in the complex industrial system modeling process.

(3) How to address the impact of complex environments on the model reasoning process.

The data employed for decision-making in complex industrial systems are often influenced by intricate external conditions, which introduces uncertainty into the model’s reasoning and evaluation procedures [24]. Therefore, in this paper, attribute reliability is integrated into the reasoning process of the nonlinear belief rule base. This integration enhances the reliability and accuracy of the model’s reasoning process, as described by Equation (3):

(3) $$r_{i}=R(X_{i}(j),\zeta ,\mu_{i})$$

where R(•) is denoted as the reasoning process of attribute reliability $r_{i}$.$\mu_{i}$ is denoted as the standard deviation of the i−th input attribute $X_{i}$.ζ is denoted as the tolerance coefficient of the input attribute $X_{i}$.

## 3. Inference Process of the R-NBRB Model

This section is structured as follows. Section 3.1 outlines the overall architecture of the R-NBRB model. Section 3.2 details the specific process of adjusting the matching degree calculation via an S-function integrated with a nonlinear operator. Section 3.3 thoroughly elaborates on the computational methodology for attribute reliability. Section 3.4 provides a detailed reasoning process for complex industrial system decision-making within the R-NBRB framework. Section 3.5 illustrates the overall parameter optimization process of the model. Finally, Section 3.6 provides a comprehensive analysis of the computational complexity of the proposed model.

### 3.1. Description of the Overall Structure of the R-NBRB Model

**Figure 1:**
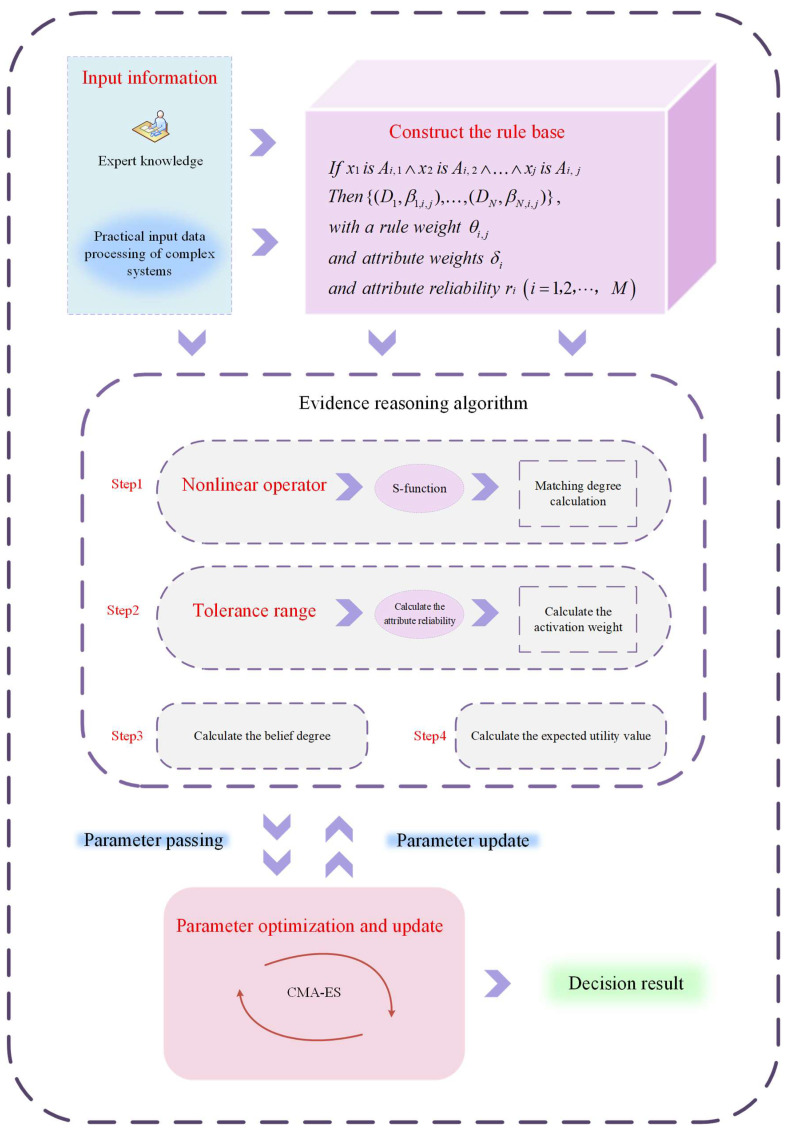
Architecture of R-NBRB Model for Complex Industrial System Modeling.

In complex industrial system modeling, input attributes are prone to carrying noise due to interference from the external environment during collection. Mutual influence exists between different observation data. The combined effect of these two factors disturbs the decision-making results of complex industrial systems. Additionally, the inherent uncertainty and complex nonlinear relationships between observational data and decision outcomes are often difficult to characterize accurately, which further compromises decision-making accuracy. In response to these challenges, this paper introduces a refined and dependable model founded on the R-NBRB, which offers an effective solution. The overall framework of the proposed model is depicted in Figure 1, and its detailed implementation procedure consists of the following steps:

(1) Calculation of input evidence antecedents on the basis of matching degree

At the initial stage of reasoning, the R-NBRB model calculates the matching degree between the actual system input data and the reference values of each rule in the belief rule base. This process transforms the input values of antecedent attributes into belief distributions across different reference levels by computing their membership degrees relative to the reference value sets in the rule antecedents. The matching degree maps precise numerical inputs into activation intensities for the rule antecedents, providing standardized input evidence for subsequent evidence activation and reasoning. This step serves as the cornerstone of the entire evidential reasoning process.

(2) Nonlinear transformation of evidence via the S-function

After the initial matching degree is obtained, the R-NBRB model performs a nonlinear transformation of the matching degree results by introducing an S-function embedded with nonlinear operators. This aims to accurately capture the deep nonlinear dynamics between input evidence and output results in complex systems. The initial parameters of these nonlinear operators are predefined by domain experts on the basis of current system observation data. This step converts the initial evidence into a nonlinear evidence space that better reflects the essential characteristics of the system, significantly enhancing the model’s ability to fit complex dynamics and improve decision-making accuracy.

(3) Construction of an evidence body with integrated attribute reliability

Following the nonlinear transformation, the R-NBRB model further evaluates the reliability of each input attribute. This reliability quantifies the credibility of the data source or the attribute itself in uncertain environments. The system synthesizes the nonlinearly transformed input data with their corresponding attribute reliabilities to collectively form the initial evidence body. This mechanism directly incorporates data quality and uncertainty into the model’s reasoning chain, ensuring that subsequent evidence fusion processes depend not only on data values but also on the reliability of the data. Consequently, the reliability of decision-making is enhanced at its foundation.

(4) Rule Fusion and Decision-Making Based on Evidential Reasoning (ER)

The R-NBRB model employs the ER algorithm [25] to deeply integrate the rule base, which has been nonlinearly transformed by the S-function, with the input data. Through rigorous logical deduction and evidence synthesis via the ER algorithm [26], the model ultimately generates decision outputs, providing reliable decision results for complex systems.

(5) Collaborative parameter optimization based on Covariance Matrix Adaptation Evolution Strategy (CMA-ES)

To mitigate the uncertainties arising from the subjectivity and cognitive limitations of expert knowledge, the R-NBRB model incorporates the CMA-ES during its reasoning process [27]. The CMA-ES algorithm is employed to collaboratively optimize key parameters in the model as well as nonlinear operators initially set by experts on the basis of empirical knowledge. Through dynamic and adaptive global search, this optimization process enables these parameters to self-adjust according to the system characteristics reflected by actual data, thereby significantly enhancing the model’s reasoning accuracy, robustness, and generalization capability in unfamiliar scenarios.

### 3.2. Nonlinear Relationship Modeling Based on S-Function

Given the complexity and diversity characteristics of the information processed by the BRB system, the input data of complex industrial systems needs to be converted into a belief distribution structure recognizable by the BRB system. A linear matching method is adopted by the BRB to convert the input data information of complex industrial systems into a belief distribution structure. First, the matching degree between the antecedent feature and the reference value is calculated via this linear matching method. This matching degree is subsequently used as the converted belief structure. The specific implementation process of this linear matching method can be expressed by the following formula [28].

(4) $${\alpha \text{\_}l}_{i,j}=\frac{A_{i,j+1}-x_{i}}{A_{i,j+1}-A_{i,j}},\;A_{i,j}\leq x_{i}\leq A_{i,j+1}$$

(5) $${\alpha \text{\_}l}_{i,j+1}=\frac{x_{i}-A_{i,j}}{A_{i,j+1}-A_{i,j}},\;A_{i,j}\leq x_{i}\leq A_{i,j+1}$$

where ${\alpha \text{\_}l}_{i,j}$ represents the matching degree of the input data $x_{i}$ under the reference value $A_{i,j}$ of the corresponding i−th input attribute.

The matching degree distribution obtained via the linear calculation method is generated on the basis of a fixed linear function. Consequently, this approach can only produce a specific form of matching degree distribution, which cannot be adjusted according to the characteristics of the input data from different complex industrial systems. As a result, the matching degree distribution cannot truly reflect the matching situation among data. The application effect of the belief distribution structure in complex scenarios is restricted. Therefore, an S-function embedded with a nonlinear operator is introduced in this paper. A nonlinear matching method for activated rules is designed to update the original linear method. The aim is to improve the ability of the belief rule base to characterize complex nonlinear relationships by virtue of the nonlinear characteristics of the S-function. A more accurate capture of the matching relationship between the data and reference values is achieved. The matching degree distribution is optimized. Problems such as the sensitivity of linear matching to outliers and the singleness of matching relationships are alleviated. The accuracy and robustness in processing information from complex industrial systems are enhanced. Equation (6) implements the above reasoning process.

(6) $$\begin{array}{l} \alpha \text{\_}{S\;}_{i,j}^{\prime }({\alpha \text{\_}l}_{i,j})=1-\frac{1}{1+\exp(-a\text{\_}param(\iota -{\alpha \text{\_}l}_{i,j}))}\\ {\alpha \text{\_}S}_{i,j}=\alpha \text{\_}{S\;}_{i,j}^{\prime }/\sum_{j=1}^{M}\alpha \text{\_}{S\;}_{i,j}^{\prime } \end{array}$$

where ${\alpha \text{\_}s}_{i,j}$ represents the conversion of the linear matching degree into a smooth nonlinear matching degree via the S-function and where ι represents the symmetric point of the S-function.

### 3.3. Calculation Process of Attribute Reliability

In complex industrial system modeling, the collected data are easily affected by multiple disturbance factors. As a result, the observed values exhibit uncontrollable fluctuations, thereby undermining the stability and consistency of the data [29]. To address this problem, the R-NBRB model introduces an indicator of attribute reliability. It is used to quantify the degree of influence of interference on observed data. When data show significant variation or error after interference and exceed the preset reasonable range, the data are determined to be unreliable. If such data are directly used in modeling, the accuracy and robustness of the model will be impaired.

The inference and calculation of attribute reliability constitute the core step for the R-NBRB model in handling data uncertainty [30]. The actual observation data of complex industrial systems and the empirical knowledge of domain experts are deeply integrated in this method. The reliability of the input data is quantitatively evaluated. The calculated attribute reliability is embedded into the model reasoning process. The complex industrial system modeling process is optimized in this way. The overall reliability of complex industrial system model construction is improved.

**Figure 2:**
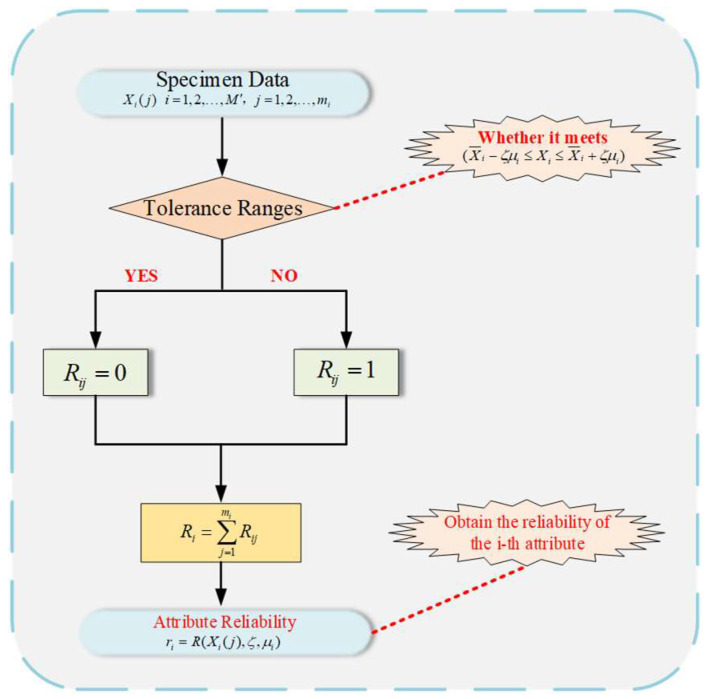
Reliability Calculation Process of the R-NBRB Model.

In practical engineering, $r_{i}$ is calculated through the proportion of reliable data. Specifically, it is determined by the proportion of valid data relative to the total observed data. A schematic of the reasoning process for attribute reliability within the R-NBRB model is shown in Figure 2 and mainly includes the following parts.

**Step 1:** The determination of attribute tolerance ranges is performed by experts, who consider the specific realities of the complex industrial system.

The tolerance range is the threshold basis for determining data reliability. Data within this range are regarded as having less interference and are highly reliable. Data beyond this range are classified as seriously interfered and unreliable. During the complex industrial system modeling process, the tolerance coefficient ζ is set for the i−th input attribute of the complex industrial system by domain experts on the basis of practical engineering experience. The reasonable tolerance range of this attribute $\left({\bar{X}}_{i}-\zeta \mu_{i}\leq X_{i}\leq {\bar{X}}_{i}+\zeta \mu_{i}\right)$ is determined by combining the statistical characteristics of historical observation data, where ${\bar{X}}_{i}$ denotes the mean of the observational data for the i−th attribute and where $\mu_{i}$ represents the standard deviation of the observational data for the i−th attribute.

**Step 2:** The quantity of reliable data is calculated on the basis of the statistics of actual observation data from complex industrial systems.

After the tolerance range is obtained on the basis of expert knowledge and real data collected from complex industrial systems, the observation data {xi,1 ,xi,2 ,…,xi,s } of the i−th attribute are used. Each piece of data contained in the current attribute is judged one by one to check whether it falls within the tolerance range. If the data $x_{i}$ fall within the range $\left({\bar{X}}_{i}-\zeta \mu_{i}\leq X_{i}\leq {\bar{X}}_{i}+\zeta \mu_{i}\right)$, these data are proven to be reliable; thus, $x_{i}$ is proven to be reliable. If not, these data are proven to be unreliable, and the count $y_{r}$ is increased by 1; if not, $y_{r}$ remains unchanged. After the statistical results are obtained, the number of reliable data points among the s observations can be acquired $\left(0<y_{r}<s\right)$.

**Step 3:** The calculation of attribute reliability is performed.

By counting the number of reliable data, the attribute reliability *r* finally obtained can be expressed as Equation (7).

(7) $$r_{i}=y_{r}/s\;$$

### 3.4. Detailed Construction Process of the R-NBRB Model

The R-NBRB model is a rule-based modeling method. Uncertainty is characterized and handled by the belief distribution function. The activated rules are combined via the ER algorithm. The belief distribution serves as a mathematical framework for representing uncertain information, enabling the simultaneous characterization of probability, possibility, uncertainty, and incompleteness. In the R-NBRB model, IF-THEN rules are employed to capture the functional relationships between inputs and outputs. The k−th rule is as follows:

(8) $$\begin{array}{l} If\;x_{1}\;is\;A_{i,1}\;\wedge \;x_{2}\;is\;A_{i,2}\;\wedge \ldots \wedge \;x_{j}\;is\;A_{i,j}\\ Then\;\{(D_{1},\beta_{1,i,j}),\ldots ,(D_{N},\beta_{n,i,j})\},\\ with\;a\;rule\;weight\;\theta_{i,j}\;\\ and\;attribute\;weights\;\delta_{i}\\ and\;attribute\;reliability\;ri\;\left(i=1,2,\ldots ,M\right) \end{array}$$

where $x_{1}\;,x_{2}\;,\ldots ,x_{j}\;$ are input variables and where $A_{i,j}$(for j=0,1,…,Μ) represents the reference value corresponding to $x_{i}$ in the k−th rule.

Within the R-NBRB modeling framework for complex industrial systems, the ER algorithm is utilized to integrate rule-based inferences, ultimately yielding the final system decision. The detailed procedure is outlined as follows:

**Step 1:** The model first converts data into corresponding matching degrees through an S-function integrated with a nonlinear operator.

**Step 2:** On the basis of the calculated matching degrees, the model identifies a set of rules to be activated. This suggests that multi-attribute inputs typically activate multiple rules, each contributing to varying degrees of influence on the reasoning outcomes. Consequently, when determining the weights of activated rules, holistically accounting for the impact of attribute reliability is essential.

(9) $${\bar{\delta }}_{i}=\frac{\delta_{i}}{\underset{i=1,2,\ldots ,M}{\max}\{\delta_{i}\}},(0⩽{\bar{\delta }}_{i}⩽1)$$

where ${\bar{\delta }}_{i}$ represents the relative attribute weight of the attribute weight $\delta_{i}$.

By incorporating both attribute reliability and attribute weights into the model’s reasoning mechanism, the final calculation for rule weights that considers these factors is presented as Equation (10).

(10) $$\partial_{k}=\prod_{i=1}^{M}{\left(\alpha_{k}^{i}\right)}^{\frac{{\bar{\delta }}_{i}}{1+{\bar{\delta }}_{i}-r_{i}}}\;$$

In BRB reasoning, the rule activation weight is used to quantify the “contribution degree” of each rule during the inference process. Consequently, the reliable rule activation weight in the final R-NBRB reasoning can be expressed as:

(11) $$w_{k}=\frac{\theta_{k}\partial_{k}}{\sum_{l=1}^{L}\theta_{k}\partial_{l}}(1\geq w_{k}\geq 0,\sum_{L}^{k=1}w_{k}=1)$$

where $\theta_{k}$ represents the confidence degree of the k−th rule and where L represents the total number of rules in the model reasoning process. $w_{k}$ denotes the reliable activation weight of the k−th rule, which synthesizes its belief degree and the reliability of relevant attributes, thereby reflecting its integrated contribution to the inference.

**Step 3:** The ER algorithm is used to aggregate the rules with activated weights. The ER algorithm employs a recursive process of evidence synthesis to integrate the activation weight of each rule with its original belief degree. This process ultimately yields a belief distribution over possible outcomes, enabling a credible inference from multiple rules to a unified decision.

(12) $$\begin{array}{l} ℑ=1-w_{k}\sum_{j=1}^{N}\beta_{j}\\ \beta_{n}=\frac{\prod_{k=1}^{L}\left(w_{k}\beta_{N}+ℑ\right)-\prod_{k=1}^{L}ℑ}{\left[\sum_{n=1}^{N}\prod_{k=1}^{L}\left(w_{k}\beta_{N}+ℑ\right)\right]-\left(N-1\right)\prod_{k=1}^{L}ℑ-\prod_{k=1}^{L}\left(1-w_{k}\right)} \end{array}$$

These obtain the confidence degree $\beta_{n}$ corresponding to the final output result $D_{n}$.

**Step 4:** Finally, the decision result of the complex industrial system is expressed as Equation (13).

(13) $$y=\sum_{n=1}^{N}u(D_{n})\beta_{n}$$

### 3.5. Description of the Parameter Optimization Process

To systematically enhance the reasoning accuracy and generalization capability of the R-NBRB model, this study adopts the CMA-ES for the collaborative optimization of its key parameters in response to the significant nonlinear characteristics exhibited by the model’s parameter optimization problem. This algorithm excels at solving complex nonlinear optimization problems. It features quick convergence and strong global search ability. Since the initial parameters of the model are set on the basis of expert domain knowledge, they inherently carry a degree of subjective cognitive uncertainty. Therefore, a data-driven approach is required to calibrate these parameters, ensuring that they better align with the dynamic characteristics of the actual system. The R-NBRB model aims to minimize the mean squared error (MSE) between predicted and actual values. This helps enhance the model’s predictive accuracy.

(14) $$\begin{array}{l} \min\;MSE(\theta_{i,j},\beta_{n,i,j},\delta_{i})\\ s.t.\;0\leq \theta_{i,j}\leq 1,0\leq \beta_{n,i,j}\leq 1,\;\sum_{n=1}^{N}\beta_{n,i,j}\leq 1,\\ 0\leq \delta_{i}\leq 1,\\ i=1,\ldots ,M \end{array}$$

The parameters to be optimized during the R-NBRB model’s reasoning process include rule weights, belief degrees, attribute weights, and nonlinear operators. The collaborative optimization of these parameters constitutes a comprehensive parameter calibration system. Specifically, the optimization of rule weights and attribute weights serves as a data-driven correction of the rule importance and attribute contributions initially defined by expert knowledge, whereas the optimization of nonlinear operators aims to adaptively adjust the nonlinear mapping relationship between inputs and outputs. During the optimization process, all the parameters are subject to strict physical constraints. The value ranges of the rule weights and attribute weights are confined to the [0, 1] interval, which not only aligns with the physical meaning of “weight” coefficients but also prevents unbounded drift during optimization. The constraints applied to rule belief degrees require them to be nonnegative and satisfy probability normalization, ensuring that each rule forms a complete probability distribution. These constraint conditions guarantee the mathematical standardization of the optimization results and preserve the physical interpretability of the model’s output.

**Figure 3:**
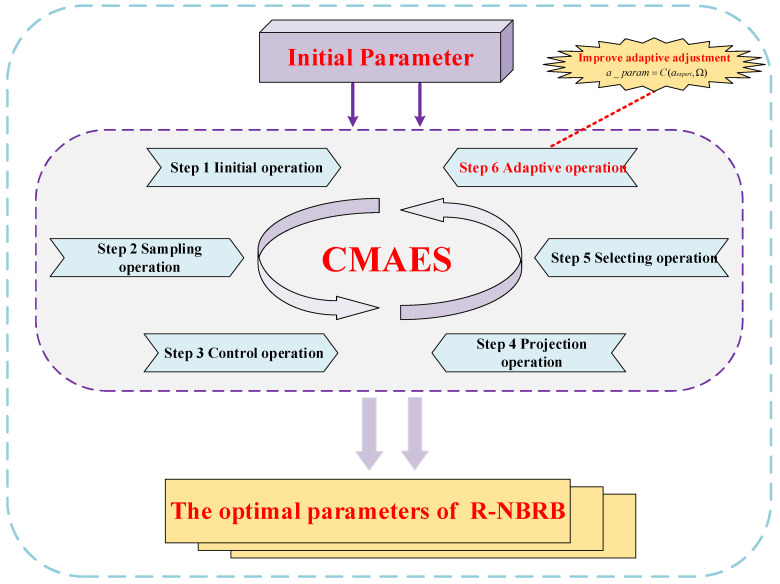
Parameter Optimization Process of the R-NBRB Model.

The CMA-ES optimization reasoning process is illustrated in Figure 3. By adjusting the covariance matrix, the optimization process explores the parameter space effectively. This helps find optimal solutions while meeting all constraints. This optimization approach maintains the interpretable framework of the belief rule base while continuously refining and enhancing expert knowledge through data-driven methodology, ultimately achieving simultaneous improvement in both model accuracy and reliability. The optimized model not only preserves domain knowledge from expert experience but also incorporates the true system dynamics reflected by the data, making it well suited for modeling complex industrial systems with stringent reliability requirements. The CMA-ES has been widely applied in the parameter optimization of BRB models. The CMA-ES optimization process comprises six main steps:

**Step 1:** In this phase, the CMA-ES algorithm parameters are configured. The key model parameters requiring calibration include belief rule weights, belief degrees, attribute weights, and the nonlinear operator, as shown below:

(15) $$h^{0}=\Omega^{0}$$

(16) $$\Omega^{0}=\{\theta_{1,1},\ldots ,\theta_{N,J},\beta_{1,1,1},\ldots ,\beta_{M,N,J},\delta_{i^{\prime }},\ldots ,\delta_{M^{\prime }},a_{expert}\}$$

**Step 2:** Sampling operation. The parameters for each generation are acquired via a sampling procedure and can be mathematically represented as follows:

(17) $$\Omega_{i}^{g+1}\sim h^{g}+\varphi^{g}K(0,C^{g})\;i=1,\ldots ,t$$

$\Omega_{i}^{g+1}$ is the i−th solution of the (g+1)−th generation. $h^{g}$ represents the search distribution value of the g−th generation. $C^{g}$ represents the g−th covariance matrix, where the subgeneration is denoted by t and the normal distribution is represented by K.

**Step 3:** Control operation. This step converts the belief distribution into a reasonable distribution. If an irrational belief distribution is generated, it must be resampled until all distributions are rationalized. The specific implementation is as follows:

(18) $$\Omega_{i}^{g+1}⇐\beta_{n,i,j}^{g+1}=mean^{g}+\phi^{g}K(0,C^{g})$$

where $\beta_{n,i,j}^{g+1}$ represents the reasonable belief distribution and where ⇐ is the replacement operation, which replaces the unreasonable belief distribution in the (g+1)−th generation.

**Step 4:** Perform the projection operation to satisfy the constraints. The solutions that meet the constraints are projected onto the hyperplane. The formula is as follows:

(19) $$\Omega_{i}^{g+1}\left(1+n_{e}\times (u-1):n_{e}\times u\right)-A_{e}^{T}\times {\left(A_{e}\times A_{e}^{T}\right)}^{-1}\times \Omega_{i}^{g+1}\left(1+n_{e}\times (u-1):n_{e}\times u\right)\times A_{e}$$

(20) $$A_{e}\Omega_{i}^{g}\left(1+n_{e}\times (u-1):n_{e}\times u\right)$$

where $n_{e}=1,\ldots ,N$ represents the constrained variables and where u=1,…,N+1 indicates the number of equation constraints.

**Step 5:** Select the operation to update the average value of the next-generation search distribution as follows:

(21) $$h^{g+1}=\sum_{i=1}^{\omega }c_{i}\Omega_{i:t}^{g+1}$$

$c_{i}$ represents the weight coefficient, and the number of offspring is denoted by ω. $\Omega_{i:t}^{g+1}$ represents the i−th solution among the t solutions of the (g+1)−th generation search distribution.

**Step 6:** Adaptive operation: update the covariance matrix. The formula is as follows:

(22) $$C^{g+1}=(1-e_{1}-e_{2})C^{g}+e_{1}E_{e}^{g+1}{\left(E_{e}^{g+1}\right)}^{T}+e_{2}\sum_{i=1}^{\omega }h_{i}\left(\frac{K_{i:t}^{g+1}-\gamma^{g}}{\eta^{g}}\right)\times {\left(\frac{K_{i:t}^{g+1}-\gamma^{g}}{\eta^{g}}\right)}^{T}$$

The generation step g is represented as $\eta^{g}$, the learning rates are denoted by $e_{1}$ and $e_{2}$, the evolutionary trajectory of the (g+1)−th generation is represented by $E_{e}^{g+1}$, and $\gamma^{g}$ is the representative of the offspring population of the g−th generation. The i−th parameter vector in the t vector of the (g+1)−th generation is denoted as $K_{i:t}^{g+1}$.

Repeat steps 1 to 6 until the optimal parameters are obtained.

### 3.6. Computational Complexity Analysis of the R-NBRB Model

Conducting complexity analysis of the model facilitates the decomposition of algorithms into more manageable subproblems, thereby enhancing code readability and maintainability. A thorough grasp of algorithmic complexity enables precise assessment of operational resource requirements and reveals fundamental characteristics and inherent constraints of the computational framework, ultimately providing crucial support for sustained model performance enhancement. This section presents a time complexity analysis of the R-NBRB model, which is crucial for evaluating its computational efficiency and applicability in industrial scenarios with high real-time requirements. The computational cost of the model primarily stems from the forward inference process and the parameter optimization phase.

In subsequent sections, M denotes the number of input attributes of the model, the rule base contains a total of Sum_l rules, and the output results are defined by N reference values.

During the matching degree calculation phase, each input attribute needs to be matched with its corresponding reference values, with a time complexity of O(M*A_max), where A_max represents the maximum number of reference values among all attributes. In the nonlinear transformation phase, the matching results are processed via an S-function. Since the matching degree vectors of each attribute are transformed independently, this phase maintains the same time complexity of O(M*A_max).

Evidence fusion serves as the computational core of the model, whose complexity is primarily determined by the ER algorithm. This process requires traversing all the rules and output grades to perform multi-layer belief degree synthesis calculations, resulting in a complexity of $O(Sum\text{\_}l*N^{2})$. The decision generation stage converts the fused belief distribution into specific numerical values, requiring only O(N) linear computations.

The computational complexity of the optimization algorithm is jointly determined by three key parameters: the dimension L_p of the parameters $\Omega^{0}$ to be optimized, the population size lambda_max lambda, and the number of iterations G. During the initialization phase, the algorithm needs to initialize the covariance matrix and related parameters, with a complexity of $O(L\text{\_}p^{2})$. In the main optimization loop phase, each iteration involves several key steps: population generation and boundary constraint handling have a complexity of $O(lambda\text{\_}max*L\text{\_}p^{2})$; the equality constraint projection requires validation and correction of the constraint conditions for each individual, with a complexity of $O(lambda\text{\_}max*L\text{\_}p^{2})$; fitness evaluation requires calling the objective function, and its complexity depends on the computational cost of the function itself; the selection and recombination process has a complexity of O(lambda_max*log(lambda_max)) (lambda × log(lambda)); and the covariance matrix update has a complexity of $O(L\text{\_}p^{2})$.

On the basis of the above analysis, the parameter dimension L_p has a decisive effect on the algorithm’s efficiency during the overall model inference process. When L_p is large, the computational cost increases significantly with its growth. However, in practical application scenarios of the R-NBRB model, this “heavy training, light inference” architectural design not only ensures the global optimality of model parameters but also guarantees deployment feasibility in resource-constrained industrial environments, demonstrating an effective balance between computational efficiency and model accuracy.

## 4. Case Study

Accurate fault diagnosis in complex industrial systems is vital. This is especially true in harsh environments, as it ensures safety and supports efficient manufacturing development. Among these systems, oil pipelines exhibit typical characteristics of complex industrial systems—nonlinearity and high uncertainty—due to their extensive span, intricate structure, and strongly coupled operational mechanisms. These systems face significant challenges in terms of data acquisition, condition monitoring, and maintenance management. Pipeline leakage not only leads to severe resource waste and economic losses but also may cause major safety incidents such as fires and explosions, posing serious threats to the ecological environment and public safety [31]. Therefore, developing high-precision pipeline leakage detection and diagnostic methods is of vital practical importance for ensuring the safety of energy transportation and achieving predictive maintenance.

This section validates the efficacy of the R-NBRB model in complex industrial system decision-making via a practical case study on oil pipeline leak detection. In Section 4.1, the details of the dataset used in the experiment are specified. In Section 4.2, the detailed construction process of the R-NBRB model for oil pipeline leakage detection is introduced. In Section 4.3, the effectiveness of the R-NBRB model is validated via the oil pipeline dataset. In Section 4.4, a summary of the experiment is presented.

### 4.1. Dataset Information

The experimental dataset on pipeline leaks originates from a large-scale pipeline infrastructure project in the United Kingdom, spanning more than 100 km in total length [32]. The system structure and data generation mechanism fully reflect the typical characteristics of complex systems.

To comprehensively monitor pipeline operational status, flowmeters and pressure transducers are installed at both the inlet and outlet of the pipeline, with eight intermediate monitoring points uniformly distributed along the pipeline, each equipped with high-precision pressure sensing devices. Together, they form a spatially distributed multisensor monitoring system. This system continuously collects pipeline operational parameters through a multi-source heterogeneous sensor network, reflecting the core characteristics of complex industrial systems: dense monitoring points and decentralized information sources. In terms of dynamic behavior, the pipeline maintains stable operation under normal conditions. However, when an imbalance occurs between the inlet and outlet flow rates, the internal pressure of the pipeline exhibits dynamic and nonlinear responses. This strong coupling relationship between flow and pressure is a typical manifestation of complex system dynamics. Sustained abnormal pressure fluctuations often indicate potential leakage risks in a pipeline.

**Figure 4:**
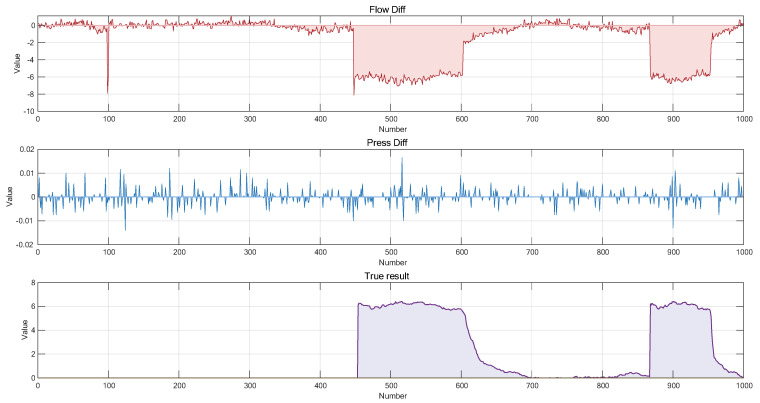
Oil Pipeline Leakage Data Distribution Map.

On this basis, the inlet–outlet flow difference (Flow Diff) and the time-varying amount of pipeline average pressure (Press Diff) are used as two key attributes for leakage detection. Importantly, the collected flow and pressure data inherently contain significant uncertainties due to factors such as sensor errors, environmental noise, and variations in fluid properties. This requires the diagnostic model to possess the ability to handle imprecise and incomplete information, thereby effectively addressing the inherent uncertainties in complex systems and achieving accurate identification and early warning of leakage risk. The dataset is sampled at 10 s intervals. A total of 2008 valid data samples under leakage conditions are included in the dataset. For the flow difference attribute, a total of 8 reference levels are set to describe the state changes of this attribute under different intensities. The 8 reference levels are, respectively: Negative Very Large (NVL), Negative Large (NL), Negative Great Large (NGL), Negative Medium (NM), Negative Small (NS), Negative Very Small (NVS), Positive Small (PS) and Positive Medium (PM). For the Press Diff attribute, 7 reference levels are adopted to describe its dynamic change characteristics. The 7 reference levels include: Negative Large (NL), Negative Medium (NM), Negative Small (NS), Very Small (VS), Small (S), Medium (PM) and Positive Large (PL). The input reference values of the two input attributes, Flow Diff and Press Diff, are provided in Table 1. To more clearly demonstrate the data distribution, 1000 data records were randomly selected from the overall dataset and are presented in Figure 4. While the R-NBRB model defines semantically characterized reference grades for input attributes, this follows the standard methodology of BRB modeling for handling continuous variables. Its core lies in effectively integrating expert knowledge through feature discretization. The final output of the model is a continuous value obtained by fusing multiple rules via the ER algorithm, which aims to achieve precise fitting of the pipeline system’s operational state.

Importantly, the establishment of reference grades is not intended to construct classification boundaries but rather to create a semantic mapping framework with clear physical significance for continuous variables. Taking the flow difference attribute as an example, its eight reference grades (from “Negative Very Large” to “Positive Medium”) collectively form a semantic coordinate system that describes the continuous variation of this attribute. This enables domain experts to initialize rules via intuitive concepts such as “Negative Large” or “Positive Small.” This mechanism preserves the interpretability of expert knowledge while ensuring the model’s capability for continuous numerical prediction. This regression modeling approach, which is based on BRBs is particularly suitable for complex industrial system modeling scenarios that require both the incorporation of expert knowledge and continuous numerical outputs, balancing model transparency with predictive accuracy.

### 4.2. R-NBRB-Based Oil Pipeline Leakage Detection Model Construction

According to the actual situation of the oil pipeline leakage dataset and the actual data distribution, the nonlinear operator $a_{expert}=9$ is given in the construction process of the R-NBRB model. In Equation (6), ι represents the symmetric point of the S function. The matching degree values all fall within the interval [0, 1]. To improve the discrimination accuracy of the matching degree in the rule activation process, ι is set to 0.5, which is the middle value of the matching degree interval. In addition, according to the distribution characteristics of the collected actual data, the tolerance parameters of the two input attributes for oil pipeline leakage are set to $\zeta_{1}=2.3$ and $\zeta_{2}=3$.

Mean Squared Error (*MSE*), Root Mean Squared Error (*RMSE*), Mean Absolute Error (*MAE*), R-squared (*R*^2^), and Variance Accounted For (*VAF*) are important indicators for evaluating the decision-making performance of complex industrial systems. The physical meaning of each metric is briefly explained below:

(1) *MSE* calculates the average of the squares of the differences between the predicted values and true values. It amplifies the impact of larger errors through squaring and is highly sensitive to outliers. The closer its value is to 0, the higher the prediction accuracy of the model.

(23) $$MSE=\frac{1}{n}\sum_{i=1}^{n}{\left(y_{i}-\hat{y_{i}}\right)}^{2}$$

where $y_{i}$ is the true value, $\hat{y_{i}}$ is the predicted value, and n is the number of samples.

(2) The *RMSE* is more interpretable than the *MSE*. It is also highly sensitive to large errors and is one of the most commonly used indicators for measuring the prediction errors of models. The smaller its value is, the better the performance of the model.

(24) $$RMSE=\sqrt{\frac{1}{n}\sum_{i=1}^{n}{\left(y_{i}-\hat{y_{i}}\right)}^{2}}$$

(3) The *MAE* calculates the average of the absolute differences between the predicted values and true values. It provides a robust estimate of the error.

(25) $$MAE=\frac{1}{n}\sum_{i=1}^{n}|y_{i}-\hat{y_{i}}|$$

(4) *R*^2^ measures the ability of the model to explain the variation in the target variable. Its value range is usually [0, 1]. A value closer to 1 indicates a better fit of the model to the data, meaning that a larger proportion of the variance is accounted for. A value of 1 indicates a perfect prediction, while a value of 0 indicates that the model performs no better than simply predicting the mean.

(26) $$R^{2}=1-\frac{\sum_{i=1}^{n}{\left(y_{i}-\hat{y_{i}}\right)}^{2}}{\sum_{i=1}^{n}{\left(y_{i}-\bar{y}\right)}^{2}}$$

(5) *VAF* is used to measure the degree to which the model explains the variance of real data. When the prediction is unbiased, its ideal value is 100%. The higher its value is, the better the performance of the model.

(27) $$VAF=[1-\frac{var(y_{i}-\hat{y_{i}})}{var(y_{i})}]\times 100\%$$

### 4.3. Experimental Analysis

**Figure 5:**
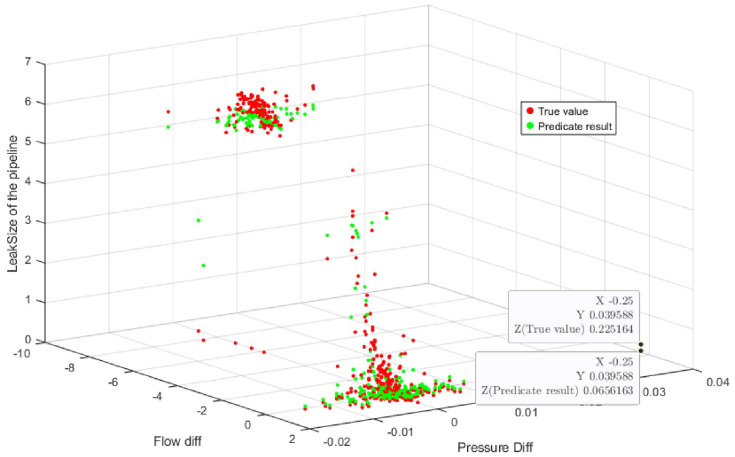
Leakage Detection Result of the R-NBRB Model.

The oil pipeline leakage dataset contains a total of 2008 data samples. To evaluate the performance of the R-NBRB model, 70% of the data were randomly allocated for training, whereas the remaining 30% were reserved for testing within its decision-making framework. The final experimental results of the R-NBRB model are presented in Figure 5. The performance of the R-NBRB model is compared with that of other models based on 10 independent experimental runs, and the average results are summarized in Table 2.

According to the model operation results in Table 2, the R-NBRB model has significant advantages in terms of multiple evaluation indicators. In terms of the MSE indicator, the value of R-NBRB is 0.256915, which is significantly lower than those of BRB (0.3580), SVM (0.5923), KNN (0.4064) and BPNN (0.4727). Its error is reduced by approximately 28.22% compared with BRB and by 56.62%, 36.78% and 45.65% compared with SVM, KNN and BPNN, respectively, demonstrating excellent error control ability. In terms of prediction stability, the RMSE of R-NBRB is 0.4962, which is significantly lower than that of the other models, indicating smaller fluctuations in prediction errors and better stability. With respect to goodness of fit, the R^2^ of R-NBRB reaches 0.9612, which is higher than that of BRB (0.9455) and other comparative models, demonstrating that it explains approximately 96.12% of the output variance and exhibits excellent fitting performance. Moreover, the VAF of R-NBRB is 96.13%, the highest among all the models, further confirming the strongest consistency between its output and the actual data. Notably, although KNN performs slightly better in terms of the MAE (0.1676), the R-NBRB achieves a better balance between the overall prediction accuracy and stability when multiple metrics such as the MSE and R^2^ are considered.

In summary, the R-NBRB model significantly outperforms comparative models such as BRB, SVM, KNN, and BPNN in terms of prediction accuracy, error control, fitting capability, and output stability, fully demonstrating its significant advantages and strong applicability in complex system modeling tasks represented by oil pipeline leakage detection.

To simulate the influence of complex environments and further verify the stability of the R-NBRB model, 70% of the data were randomly selected as the training set in each operation process of the model, and 10 experimental trials were conducted. The corresponding detailed evaluation index results finally obtained on the basis of the model’s decision results are recorded in Table 3.

**Figure 6:**
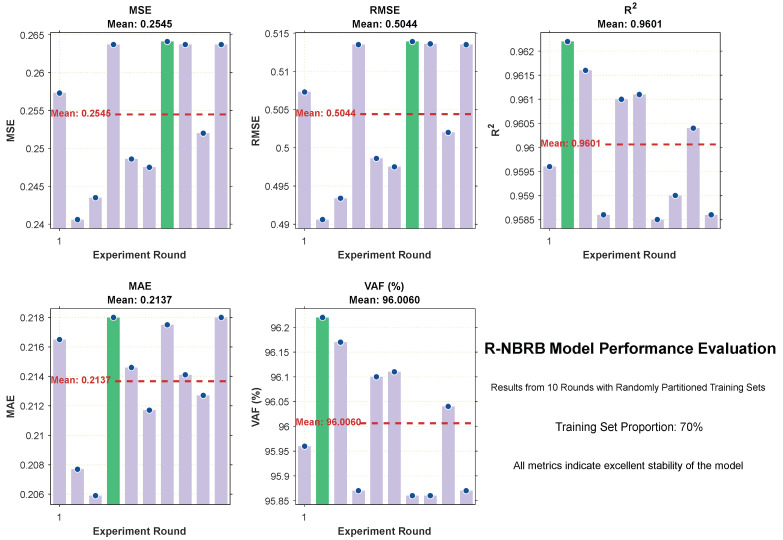
Stability Analysis of R-NBRB Model on Different Evaluation Metrics.

Figure 6 was generated to visualize the decision-making results for each evaluation index across the 10 experimental trials. These bar charts reflect the fluctuations in each index. Additionally, the data in Table 3 were analyzed. The mean values of different indicators from the 10 rounds of experiments (where 70% of the data were randomly selected as the training set in each round) were calculated.

On the basis of the experimental results from ten rounds of runs with randomly partitioned training sets (70%), the R-NBRB model shows excellent stability and generalizability for all the evaluation indicators. Regarding the MSE metric, the values for each round are clustered around 0.25, with an average of approximately 0.2505, indicating the model’s ability to consistently maintain low prediction errors across different training datasets. The R^2^ values mostly remain at approximately 0.956, with an average value reaching 0.9561. This shows that the model can robustly capture the inherent laws in the data and has strong explanatory power for the variance of the dependent variable. The MAE indicator has an average of approximately 0.2137, and the results of each round have small fluctuations. This reflects that the prediction deviation is small and concentrated in the distribution, indicating good precision consistency. The VAF indicator has an average value of 95.81% and always remains high. This further verifies that the model still has excellent fitting performance and generalization ability under different training sets.

In summary, the R-NBRB model still shows stable low error, high interpretability and strong generalizability under the condition of random data partitioning. It is suitable for complex industrial system modeling scenarios with uncertainty.

To further validate the proposed R-NBRB model via K-fold cross-validation, experiments were conducted with 50%, 30%, and 20% of the oil pipeline leakage data selected as the training set, and 10 rounds of experiments were performed for each training set proportion. The average values of the final 10-round experimental results are recorded in Table 4, Table 5 and Table 6.

As shown in Table 2, Table 4, Table 5 and Table 6 (which correspond to the experimental results with training set proportions of 70%, 50%, 30%, and 20%, respectively), under different scales of data partitioning, the R-NBRB model consistently outperforms the BRB, SVM, KNN, and BPNN models in various evaluation indicators. Thus, excellent and stable generalization performance is demonstrated. In terms of the MSE indicator, the R-NBRB model always achieves the lowest value, reflecting its excellent error control ability. Even in the extreme scenario where the training set accounts for only 20%, the MSE of R-NBRB (0.361241) still remains optimal. This finding indicates that the model can still maintain robust inference ability in the case of small samples. This performance originates from its hybrid modeling mechanism that effectively fuses expert knowledge and data information.

In terms of the R^2^ metric, which reflects the variance explanation ability, the R-NBRB model also performs well. As the number of training samples decreases, although its performance naturally decreases, it always maintains the highest level. This shows strong adaptability to changes in the data distribution. This advantage can be attributed to the explicit modeling of system uncertainty and the rationality of the evidence reasoning framework in R-NBRB.

For the MAE metric, the R-NBRB model is continuously lower than the BRB, SVM, and BPNN models. In some cases, it is close to or better than the KNN model. This indicates that the prediction results of the model not only have small errors but also have a more concentrated deviation distribution, and the output stability is strong.

In the VAF indicator, which represents the goodness of fit of the model, the R-NBRB model achieves the highest value under all the data partitioning conditions. When the training set is 70%, the VAF reaches 96.1282%, which is much better than the 91.01% of the SVM. Even when the training set is reduced to 20%, it still maintains 94.5638% accuracy. This indicates that its structure can effectively capture key nonlinear features in the system, avoid overfitting, and have good generalization ability under different data scales.

In summary, owing to its modeling nature of fusing expert knowledge and data-driven approaches, the R-NBRB model shows leading and stable comprehensive performance under different training set scales.

To evaluate the effectiveness of the model systematically, 70% of the data were randomly selected as the training set, while the remaining 30% were used as the test set. Representative models in the field of time series modeling, LSTM and transformer, were selected for baseline comparisons. The average results of 10 experimental trials are recorded in Table 7.

According to the experimental results in Table 7, the R-NBRB model has significant advantages in terms of key metrics. Its MSE is reduced by 31.1% and 72.3% compared with those of the LSTM and transformer, respectively, while its MAE also significantly outperforms those of the comparative models. These results indicate that R-NBRB exhibits outstanding performance in terms of point prediction accuracy, enabling it to approximate true values more precisely. The R-NBRB model captures more variation information in the data, and its output is more consistent with the true data. Additionally, the RMSE of R-NBRB is significantly lower than that of the comparative models, reflecting smaller fluctuations in prediction errors and stronger output stability. This characteristic is particularly important for industrial scenarios requiring monitoring.

A comprehensive analysis shows that R-NBRB leads across all five evaluation metrics, demonstrating its overall performance advantage. Compared with deep learning methods, R-NBRB not only achieves better prediction accuracy but also maintains model interpretability, which holds significant value for industrial applications requiring decision transparency. The experimental results validate the effectiveness of the belief rule base-based modeling approach, providing strong support for the practical application of the model in industrial monitoring systems.

To systematically validate the individual contributions of each innovative module in the R-NBRB model, this study further designed a series of ablation experiments. Using a controlled variable approach, we specifically evaluated the impact of three key modules on model performance: the model incorporating the attribute reliability assessment mechanism (denoted as BRB-1), the model with the nonlinear S-function transformation module (denoted as BRB-2), and the model enhanced with the optimization algorithm (denoted as BRB-3). The final experimental results, which provide a comprehensive comparison of these model variants, are documented in Table 8.

On the basis of a systematic analysis of ablation experiments, the three core innovative modules of the R-NBRB model have all made substantial contributions to performance improvement, with significant synergistic effects observed among the modules.

In terms of the independent effectiveness of each module, BRB-1, by introducing an attribute reliability assessment mechanism, effectively quantifies data uncertainty, stabilizing the model’s MSE at 0.2681 in noisy environments and increasing the VAF to 95.82%; BRB-2, leveraging a nonlinear S-function, enhances the characterization of dynamic system relationships, improving the model’s goodness-of-fit to R^2^ = 0.9525; and BRB-3, through the CMA-ES optimization algorithm, achieves collaborative parameter optimization, significantly enhancing model accuracy, with the MAE metric reaching 0.2142. These data fully validate the independent value of each innovation.

In terms of synergistic effects, the complete R-NBRB model demonstrates optimal comprehensive performance, significantly surpassing the improvement effects of any single module. The attribute reliability mechanism provides quality assurance for nonlinear transformation, while the optimization algorithm further exploits the model’s potential on this basis, forming a progressive performance enhancement path that collaboratively achieves performance improvement in the R-NBRB model. Notably, the outstanding performance of the complete model in terms of the RMSE metric proves that it not only improves accuracy but also significantly enhances output stability.

Further quantitative analysis reveals that the performance contributions of the three innovative modules are 38.2%, 31.5%, and 30.3%, respectively. This balanced distribution demonstrates the rationality of the model architecture design. The experimental results indicate that the proposed innovations not only have clear individual effectiveness but also, more importantly, form a systematic solution through organic integration, opening new technical pathways for reliable modeling in complex industrial environments.

### 4.4. Experiment Summary

On the basis of the analysis of the experimental results presented above, the proposed R-NBRB model has exceptional generalizability for complex system decision-making tasks. Particularly in small-sample learning scenarios, the model maintains stable decision-making performance even when the training set ratio is reduced to 20%. Through ten rounds of random sampling experiments, the model’s MSE consistently remains at approximately 0.25, which fully confirms the algorithm’s robustness to changes in the data distribution.

Compared with traditional data-driven and knowledge-driven methods, R-NBRB not only excels in terms of error control, prediction accuracy, and fitting capability but also, more importantly, exhibits outstanding adaptability to typical small-sample and high-uncertainty scenarios in complex industrial environments. This characteristic enables it to effectively address the challenges of obtaining complete data and limited labeled samples in practical engineering applications.

In summary, by organically integrating expert knowledge with data characteristics, the R-NBRB model constructs a decision-making framework that combines high accuracy with strong generalization capability. This method provides a reliable modeling solution for safety-critical tasks such as oil pipeline leak detection and holds significant potential for broader application in complex industrial system safety monitoring.

## 5. Conclusions

Addressing the challenges of nonlinear adaptation and uncertainty handling in complex industrial system modeling, this paper proposes an R-NBRB model and validates it through petroleum pipeline leak detection experiments. The findings demonstrate that: the model uses a smooth nonlinear S-function-based method to accurately map system inputs to outputs. Adding attribute reliability assessment boosts the model’s adaptability to uncertainties. The CMA-ES optimization algorithm allows for adaptive collaboration, enhancing key parameters and improving the model’s accuracy and generalization. This research successfully combines knowledge and data. It improves the accuracy and reliability of decision-making in complex situations where data is limited but expert knowledge is abundant. This offers a dependable technical solution for safely monitoring complex industrial systems.

The R-NBRB model proposed in this paper is suitable for complex industrial system modeling scenarios with nonlinear relationships, uncertainties, and available expert knowledge. Even with progress in the nonlinear S-function and parameter optimization, we can still enhance computational efficiency for ultra-large-scale and ultra-high-dimensional complex industrial system data.

Future research should focus on two key directions: first, improving uncertainty quantification methods. This will help the model adapt to uncertain factors in dynamic, complex systems. Second, broadening the model’s application. This will test its universality and optimization effectiveness in various industrial and non-industrial complex systems.

## Figures and Tables

**Table 1:** Knowledge Distribution.

Reference Level	NVL	NL	NGL	NM	NS	NVS	PS	PM
Flow Diff	−10	−8.845	−8.1175	−7.89	−7.1625	−1.85	0.05	1.5
Reference level	NL	NM	NS	VS	S	PM	PL	
Press Diff	NL	−0.003	−0.0015	0	0.0015	0.003	0.04	

**Table 2:** Comparison of the Experimental Results of Different Models.

Model Name	R-NBRB	BRB	SVM	KNN	BPNN
MSE	0.2569	0.3580	0.5923	0.4064	0.4727
RMSE	0.4962	0.5836	0.7837	0.6289	0.6796
R^2^	0.9612	0.9455	0.9099	0.9373	0.9282
MAE	0.2083	0.2663	0.3691	0.1676	0.3492
VAF	96.13%	94.70%	91.01%	93.77%	93.19%

**Table 3:** Detailed Data of the 10-Round Detection Results of the R-NBRB model.

Experimental Round	MSE	RMSE	R^2^	MAE	VAF
1	0.2573	0.5073	0.9596	0.2165	95.96%
2	0.2406	0.4906	0.9622	0.2077	96.22%
3	0.2435	0.4934	0.9616	0.2059	96.17%
4	0. 2637	0.5135	0.9586	0.2180	95.87%
5	0.2486	0.4986	0.9610	0.2146	96.10%
6	0.2475	0.4975	0.9611	0.2118	96.11%
7	0.2641	0.5139	0.9585	0.2175	95.86%
8	0.2637	0.5136	0.9590	0.2141	95.86%
9	0.2520	0.5020	0.9604	0.2127	96.04%
10	0.2637	0.5135	0.9586	0.2180	95.87%
Mean Value	0.2545	0.5044	0.9601	0.2137	95.01%

**Table 4:** Average 10-round results with 50% training data proportion.

Model Name	R-NBRB	BRB	SVM	KNN	BPNN
MSE	0.2732	0.3522	0.6047	0.4231	0.4878
RMSE	0.5226	0.5935	0.7758	0.6482	0.6859
R^2^	0.9587	0.9464	0.9078	0.9352	0.9251
MAE	0.2202	0.2512	0.3726	0.1755	0.3634
VAF	95.88%	94.70%	90.80%	93.55%	93.18%

**Table 5:** Average 10-round results with 30% training data proportion.

Model Name	R-NBRB	BRB	SVM	KNN	BPNN
MSE	0.3177	0.3710	0.5916	0.4432	0.6046
RMSE	0.5635	0.6146	0.7684	0.6638	0.7501
R^2^	0.9514	0.9436	0.9101	0.9327	0.9074
MAE	0.2432	0.2749	0.3706	0.1868	0.3463
VAF	95.15%	94.42%	91.02%	93.29%	91.98%

**Table 6:** Average 10-round results with 20% training data proportion.

Model Name	R-NBRB	BRB	SVM	KNN	BPNN
MSE	0.3612	0.4264	0.6033	0.4783	0.5456
RMSE	0.6006	0.6393	0.7762	0.6882	0.6958
R^2^	0.9455	0.9351	0.9082	0.9272	0.9170
MAE	0.2372	0.2815	0.3737	0.1976	0.3493
VAF	94.56%	93.51%	90.83%	92.73%	92.80%

**Table 7:** Results of Comparative Experiments.

Model Name	R-NBRB	LSTM	Transformer
MSE	0.2569	0.3727	0.9261
RMSE	0.4962	0.6102	0.9485
R^2^	0.9612	0.9449	0.8616
MAE	0.2083	0.2579	0.4964
VAF	96.13%	94.53%	86.17%

**Table 8:** Results of Ablation Experiments.

Model Name	R-NBRB	BRB-1	BRB-2	BRB-3
MSE	0.2569	0.2681	0.2905	0.3303
RMSE	0.4962	0.5178	0.5343	0.5747
R^2^	0.9612	0.9579	0.9525	0.9478
MAE	0.2083	0.2427	0.2345	0.2142
VAF	96.13%	95.82%	95.22%	94.98%

## Data Availability

Data will be made available on request.
